# Treatment of Purpura

**Published:** 1876-09

**Authors:** 


### TREATMENT OF PURPURA.

The interest in Dr. Bulkley's paper centered upon the use of ergot hypodermically in the treatment of that affection. He preferred the fluid extract to ergotine. The injections were introduced by preference about the pectoral muscles or sides of the chest. Large doses were required, and min. x. or xv. or even xx. or xxx. might be repeated every hour and a half. The remedy might also be given by the mouth, and he related a case in which he used oz. ij. of the fl. ext. within ten days, administered in half-drachm doses. Dr. Bulkley also made some remarks upon hemorrhagic small-pox, chiefly relating to the differential diagnosis between it and measles.

The President called Dr. Kendall, of Onondago county, to the Chair.

The paper being open for discussion, Dr. Squibb remarked that he wished to say a single word of caution with reference to the size of the dose of the fl. ext. of ergot. Dr. Bulkley had recommended that ounce doses of the fl. ext. might be administered without harm, but that must be exceptional. He should be very sorry to have the paper go out from this society without some check as regards such a dose as that. He believed that ergot, like very many other drugs, was liable to tolerance, but to make the broad statement that in ordinary cases the fl. ext. could be administered in ounce doses had a large element of danger in it. Papers presented there were not like papers presented for publication in the Journals. They stood then upon the indorsement of the Society; and if they went out in the Transactions, they went out with its indorsement.

Dr. Castle, of New York, directed attention to the fact that the injection in Dr. Bulkley's cases had been made in the gluteal and posterior scapular regions; and he was of the opinion that a much more favorable locality was the anterior wall of the abdomen, on account of the less liability to wound important nerves and blood vessels, and in case abscess should be formed, it would not give the patient so much inconvenience as if situated in either of the localities mentioned.

Dr. Bulkley remarked, with regard to the size of the dose referred to by Dr. Squibb, that he recommended from min. x. to xv. of the fl. ext., and only exceptionally a half drachm. The statement with regard to half and whole ounce doses, was according to Dr. H. C. Wood, of Philadelphia; but Dr. Bulkley did not embody it in his paper, did not regard it as his own statement, and should be sorry to have it impair the value of the paper.

With regard to the criticism of Dr. Castle, Dr. Bulkley remarked, he recommended that the injection should be introduced somewhere about the soft parts of the pectoral muscles or sides of the body. It was in a case which Dr. Hanks had treated, and to which Dr. B. had referred, that the injections were made in the gluteal region. Cleanliness of the syringe, slowness of administration, and depth of insertion, were regarded as points of importance, if the best effects would be realized from the remedy, and abscesses avoided. Dr. B. regarded abscesses as of infrequent occurrence if the injections were properly given.

Dr. Cartwright, of Delaware county, asked if ergot was so valuable in the treatment of various hemorrhages and spinal congestion, why it would not be serviceable in the treatment of congestion of the brain, especially in congestive apoplexy?

Dr. Squibb regarded Dr. Bulkley's experience with reference to abscesses following the use of ergot hypodermically as unusually favorable, and thought that there were gentlemen present who had had a very different experience. He did not believe that Dr. Bulkley meant to incorrectly represent the result of using the remedy in that manner when he spoke of the general harmlessness of the injections; but his experience, and he believed that of others, did not justify such conclusion.

Dr. Wey, of Elmira, remarked that formerly abscess almost invariably followed his use of ergot hypodermically; but latterly, since he had used Dr. Squibb's aqueous solution, and used it only of such strength that *one minim represented one grain*, no more, he had not seen a single abscess produced, and the effect was just as decided.

Dr. Squibb remarked that the aqueous extract was much less irritating than the ordinary fluid extract, and further remarked that with regard to effect produced, it was best to gradually increase the dose until uterine colic was produced, and then fall a little back of that point, when the drug could be continued.

Dr. Stoddard, of Rochester, remarked that he had seen one drachm of the fluid extract introduced into the cavity of the uterus by Dr. Dean, of that city, and the physiological action of the drug produced in half an hour in most cases, and the effect was no more severe than when min. xv. or xx. were used hypodermically.

He had not seen any ill effect follow the introduction of the drug into the substance of the uterus itself, and had seen employed for that purpose Squibb's solid extract in the strength of one grain to ten minims of water.

Dr. Wey remarked that the largest dose of ergot he had employed was that in which one drop represented four grains of the powdered ergot; and the barrel of an ordinary hypodermic syringe had been filled and thrown into the connective tissue day after day without unpleasant symptoms other than the formation of abscess.

Dr. Squibb remarked, with reference to poisoning by ergot, that there was no evidence of the existence of such symptoms as were detailed in the older text-books. He had repeatedly known of drachm doses being administered three or four times a day without rendering patients uncomfortable in any such sense as being toxic.—*Medical Society State of New York.*—*Medical Record.*

